# Is it time for a paradigm shift in understanding embryo selection?

**DOI:** 10.1186/1477-7827-13-3

**Published:** 2015-01-11

**Authors:** Norbert Gleicher, Vitaly A Kushnir, David H Barad

**Affiliations:** The Center for Human Reproduction, 21 E 69th Street, New York, NY USA; The Foundation for Reproductive Medicine, New York, NY USA

## Abstract

**Background:**

Embryo selection has been an integral feature of in vitro fertilization (IVF) almost since its inception. Since the advent of extended blastocyst stage embryo culture, and especially with increasing popularity of elective single embryo transfer (eSET), the concept of embryo selection has increasingly become a mainstay of routine IVF.

**Discussion:**

We here, however, argue that embryo selection via blastocyst stage embryo transfer (BSET), as currently practiced, at best improves IVF outcomes only for a small minority of patients undergoing IVF cycles. For a large majority BSET is either ineffective or, indeed, may actually be harmful by decreasing IVF pregnancy chances. Overall, only a small minority of patients, thus, benefit from prolonged embryo culture, while BSET, as a tool to enhance IVF outcomes, is increasingly utilized as routine care in IVF for all patients.

**Summary:**

Since newer methods of embryo selection, like preimplantation genetic screening (PGS) and closed system embryo incubation with time-lapse photography are practically dependent on BSET, these concepts of embryo selection, currently increasingly adopted in mainstream IVF, require reconsideration. They, automatically, transfer the downsides of BSET, including decreases in IVF pregnancy chances in some patients, to these new procedures, and in addition raise serious questions about cost-effectiveness.

## Background

As delivery of a single healthy child is increasingly perceived as the only desired outcome for in vitro fertilization (IVF), the concept of embryo selection (ES) has gained ever-greater popularity.

ES is not a new concept; it has been an essential part of IVF practice almost since the inception of IVF. The search for best embryos (in the past), and the search for *the* best single embryo overall (now) in association with elective single embryo transfer (eSET) has always been a "holy grail" of IVF. Recent experiences with ES, however, raise serious questions about the validity of the concept, as currently proposed and increasingly integrated by many IVF centers all over the world into routine clinical IVF practice.

Though already considered before as a concept [1, 2], ES for the first time significantly affected routine IVF with introduction of blastocyst stage embryo transfer (BSET), when Schoolcraft’s groups reported that implantation and clinical pregnancy rates after BSET appeared superior to cleavage-stage embryo transfers (CSET) [3, 4].

The utilization of BSET has, since, made increasing inroads into IVF, as more and more IVF centers switched from day-3 cleavage stage to routine day-5/6 transfers of blastocyst-stage embryos. BSET received further support, when recently an allegedly improved method of preimplantation genetic screening (PGS), utilizing trophectoderm biopsy (an additional, secondary method of ES), was proposed as part of routine IVF, and quickly found supporters [5, 6].

BSET and this new form of PGS are, however, exactly why the general concept of ES, as discussed below, requires careful reconsideration.

## Discussion

BSET has to be the starting point for such a discussion. It is based on the seemingly logical concept that longer embryo culture *in vitro* favorably selects best embryos, while poorer quality embryos will arrest on the way to culture days-5/6. As before noted, BSET was popularized by the work of Schoolcraft’s group’s [3, 4], but was followed up by studies of other investigators, which strongly supported the concept [7–9].

Despite almost universal acceptance of the hypothesis that BSET improves pregnancy chances in association with IVF, available published data suggest that this hypothesis, at least in part, is actually false. The reason is that BSET improves pregnancy rates in association with IVF *only* in "good-prognosis" patients.

While this statement probably will come as a surprise to most readers of this debate article, this opinion is based on rather well supported metaanalyses: In a Cochrane Database Systemic Review of 18 randomized clinical trials, comparing CSET and BSET, Blake et al. were the first to report live birth rates significantly favoring BSET but only for "good-prognosis" patients [10]. Table 1 summarizes in detail the findings of this study.

**Table 1:** **Outcome summary of metaanalysis of CSET vs. BSET by Blake et al.** [10]*

Study finding	OR	95% CI
Improved live birth rate (36.0% vs 29.4%) favoring BSET However, only in good prognosis patients, and only when randomization on day-3;	1.35	1.05 to 1.74
Improved embryo freezing rates with CSET	0.45	0.36 to 0.56
Absence of at least 1 embryo for transfer more frequent with BSET (8.9% vs. 2.8% of cycles), though in good prognosis patients there was no such difference**	2.85	1.97 to 4.11

*18/50 identified trials met randomization (RCT) criteria, and were included.

**Accounts at least partially for BSET’s live births benefits only in "good prognosis" patients.

The authors concluded that the study provided evidence of significant difference in pregnancy and live birth rates in favor of BSET in "good-prognosis" patients, with high numbers of 8-cell embryos on day-3 being the most favored subgroup, and also being the only sub-group demonstrating no difference in cycle cancellation rates because of absence of transferrable embryos [10].

Glujovsky et al., five years later in 2012, published another Cochrane metaanalysis on the subject, with results summarized in Table 2 [11]. They added five studies to the prior Cochrane review by Blake et al. [10] and, as Table 2 demonstrates, basically confirmed their prior conclusions. They, however also added a very important additional analysis of cumulative pregnancy rates from single embryo cohorts, obtained during one retrieval, and either transferred by CSET or BSET.

**Table 2:** **Outcome summary of metaanalysis of CSET vs. BSET by Glujovsky et al.** [11]*

Study finding	OR	95% CI
12 RCTs (1550 women) significantly higher live births favoring BSET of 38.8% vs. 31.0%	1.40	1.13 to 1.74
Clinical pregnancy rates, however, did not differ (41.6% vs. 38.6%)	1.74	0.99 to 1.32
No difference in miscarriage rates	1.18	0.86 to 1.60
4 RCTs (266 women) significantly improved cumulative pregnancy rates favoring CSET (56.5% vs. 46.3%)	1.58	1.11 to 2.25
11 RCTs (1729 women) rates of embryo freezing significantly higher with CSET	2.28	2.35 to 3.51
16 RCTs (2495 women) absence of at least 1 embryo for transfer was significantly higher with BSET (8.9% vs. 3.34%)**	0.35	0.24 to 0.51

*23/50 identified trials met randomization (RCT) criteria, 5 more than in Blake study [10] and Table 1.

**Accounts at least partially for higher cumulative pregnancy rate with CSET.

These authors concluded that, overall, their study provided evidence of a small but significant difference in live birth rates favoring BSET. However, cumulative pregnancy rates from CSETs, including fresh and thaw cycles, significantly, and to a substantial degree, exceeded those of BSETs. They further concluded that this difference, likely is explained by higher rates of frozen embryos and lower failures to transfer at least one embryo.

**Figure 1:**
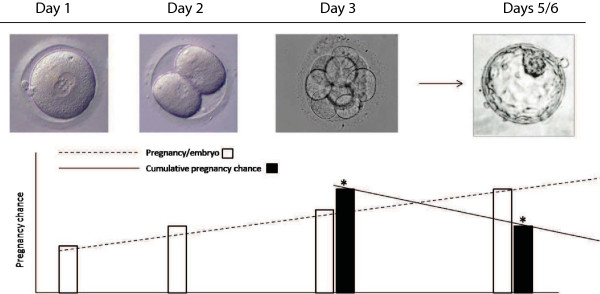
**Schematic scheme, demonstrating increasing pregnancy/delivery rates and declining cumulative pregnancy/delivery rates with lengthening embryo culture.** The figure demonstrates with prolonged embryo culture increasing pregnancy/delivery rates per embryo transfer (white bars), and declining cumulative pregnancy/delivery rates for whole embryo cohorts (black bars). This discrepancy, likely, reflects the loss of potentially viable embryos with normal pregnancy/delivery potential during prolonged embryo culture. For further detail see text.

Figure 1 describes schematically the conclusions one has to reach from these two Cochrane studies: They suggest that only so-called "good-prognosis" patients really benefit from BSET. While there is no consensus in the literature on how "good-prognosis" can or should be defined, Blake et al. convincingly demonstrated in their analysis that this means a relatively large number of high quality embryos on day-3 after fertilization is required for such a definition [10]. Considering that both studies also suggest that the number of cancelled cycles and of frozen embryos is predictive of IVF outcomes [10, 11], it would appear reasonable to assume that a patient with truly "good-prognosis" should have at least six, but maybe as many as eight, high quality embryos on day-3.

One has to further conclude that how many embryos define "good-prognosis," will change with advancing female age since implantation rates per embryo decline. In practical terms this means that, with advancing age, increasingly larger day-3 embryo numbers will be required to establish a patient’s "good-prognosis" status. Yet, embryo numbers decline with advancing age.

The ultimate conclusion from all of these considerations, therefore, has to be that, with reasonable certainty, only young women with more than 6–8 high quality embryos on days-3 will benefit from BSET.

But even young women with large embryos numbers will benefit only if their goal is to achieve pregnancy in the quickest possible way. If their goal is to maximize their cumulative pregnancy and delivery chances from all of their available embryos, then even young women should only undergo CSETs.

What percentage of "good-prognosis" patients a center serves will, of course, depend on a center’s patient population but also how a given center stimulates patients since intensity of ovarian stimulation affects available embryo numbers on day-3. How many "good-prognosis" women will favor rapid conception over best cumulative outcome chances may also vary between centers, and will, likely, depend on the quality of the informed consent.

Combined, the two studies by Blake at al [10] and Glujovsky et al. [11], therefore suggests that women with average prognosis, likely will not benefit from BSET, and only a relative small minority of IVF patients will really benefit from BSET. Moreover, especially "poor-prognosis" patients, usually characterized by low functional ovarian reserve (LFOR), run a significant risk of being harmed by BSET since they are at highest risk with prolonged embryo culture of not reaching embryo transfer for lack of transferrable embryos [11].

All of these conclusions confirm professional opinions, already previously expressed by some investigators [12, 13]. Their opinions and the findings of both Cochrane studies can only be explained if some cleavage-stage embryos result in pregnancies and healthy deliveries if transferred into the uterus on days-3 but fail to survive prolonged *in vitro* culture to blastocyst stage, This conclusion contradicts opinions by other investigators that embryos which do not make it to blastocyst stage also already at cleavage stage lack pregnancy potential [14, 15]. No other possible scenario, however, explains why cumulative pregnancy rates from embryos transferred at cleavage stage (day-3) so significantly outperform transfers at blastocyst stage (days-5/6) [11].

In clinical practice the difference in cumulative pregnancy chances between BSET and CSET creates obvious dilemmas, requiring in some patients choices between better immediate or better cumulative pregnancy chances. This dilemma, however, only exists in IVF cycles with large embryo numbers, where ES may make sense to maximize immediate pregnancy chances. One group of patients who immediately comes to mind is, of course, young women with polycystic ovary syndrome (PCOS).

Women with LFOR, who usually produce small egg and embryo numbers, should *not* be cultured to blastocyst stage. This, of course, mostly includes older women but also younger patients with premature ovarian aging (POA).

Current absence of reliable age-specific data as to what represents "good-prognosis" patients represents a potential clinical dilemma. Since this definition, as noted before, is to a significant degree center-specific, it would appear appropriate to statistically define "good prognosis" at every program individually based on embryo numbers on day-3. As also, noted before, since embryo implantation rates decline with advancing female age, this number will have to be age-adjusted.

Cost effectiveness considerations create a second dilemma because prolonged embryo culture adds cost to an already expensive IVF procedure. We have been unable to find specific data on additional costs from BSET in the literature; but two days of additional embryo culture, of course, have to incur significant additional staff, media, equipment and space costs. Moreover, young women with normal ovarian reserve and large day-embryo numbers will, most likely, also achieve excellent pregnancy and delivery rates with CSET. Whether a small incremental gain in pregnancy and delivery chances, therefore, in such patients is cost-effective, also still remains to be determined.

Even though BSET is gaining increasing acceptance in routine clinical IVF, both of these very obvious clinical dilemmas point out that this surge in utilization of extended embryo culture has been occurring without proper definition of suitable patient populations. The unavoidable conclusion, therefore, is that among women currently undergoing routine BSET, a large majority does so without gaining clinical benefits, and some even with harm to their pregnancy chances.

Further considerations of cost-effectiveness of BSET in good prognosis patients also have to take into account that BSET is increasingly combined with eSET. Whether eSET or two-embryo transfer should be routine in IVF, and at which ages, has in itself, remained a still controversial issue, and exceeds the framework of this manuscript. All of these issues, however, well demonstrate the multifactorial nature of a decision-making process that is required in devising appropriate IVF strategies.

Trisha Greenhalgh, a leading expert on evidence-based medicine and colleagues form the Evidence Based Medical Renaissance Group, recently pointed this issue out in severely criticizing how evidence-based medicine is currently practiced [16]. We strongly recommend to readers of this opinion piece to read Greenhalgh’s manuscript in its entirety. It is really remarkable how much all of those authors’ criticisms apply to the subject of ES, as currently practiced in association with IVF, and, addressed here.

Which brings us to PGS, which in its recent reintroduction to IVF is performed by utilizing trophectoderm biopsy of embryos at blastocyst stage. The hypothesis behind PGS assumes that further ES can be obtained by selecting out euploid embryos for transfer [7]. By relying on BSET after trophectoderm biopsy of day-5/6 embryos, PGS, of course, automatically subjects itself to all of above raised concerns about BSET. One, therefore, has to conclude that PGS should only be considered in "good-prognosis" patients [17].

Because the additional cost of PGS is far in excess to that of prolonged embryo culture, above noted two clinical dilemmas apply to PGS to an even more significant degree. The cost-effectiveness of BSET in association with PGS in "good-prognosis" patients is, therefore, even more questionable than with BSET alone. Potentially troubling further doubts about PGS as a method of ES were recently raised by Casper’s group in Toronto, who questioned the reproducibility of trophectoderm biopsy results in an ESHRE presentation, which attracted considerable attention [18].

Finally, these questions about ES also raise doubts about recently marketed automated closed embryo culture and time-lapse photography systems, claiming ES benefits. The primary claim of these systems is that they maximize embryo selection and subsequent BSET. A recently published comprehensive review of the literature concluded that these platforms have the potential of revolutionizing embryology; but the authors also concluded that currently available data have so far failed to demonstrate outcome benefits [19].

Following publication of this review, a first randomized study of such a system reported mild outcome benefits for the system. Importantly, the authors, however, acknowledged that their conclusions (again) were only applicable to very "good- prognosis" patients [20]. Despite marginal outcome improvements in "good-prognosis" patients, this study, therefore, again raises questions about utility of ET in only a relatively small group of "good-prognosis" patients, and about cost-effectiveness, especially considering that costs of currently marketed automated embryo culture systems in the U.S. exceed $100,000.

### Summary

All of these considerations create significant concern about the rapidly and uncontrolled utilization of ES in routine IVF. Our concern is particularly directed at the fact that patients are asked to pay for ES, while a majority among them, likely, will either gain no outcome benefits or actually face reduced pregnancy chances.

Considering that in 2012 (the last year national data are available for) in the U.S. alone almost 200,000 IVF cycles were performed, and worldwide approximately 1.5 million, the ethical and economic dimensions of here outlined problems appear obvious.

Particularly troublesome is the indiscriminate application of ES in women of all ages, and independent of FOR, when treatment decisions really should be individualized, based on the best benefits for patients [16]. Currently utilized methods of ES, at minimum, therefore need to be carefully reevaluated under objective study conditions, considering patients’ ages and their degree of LFOR.

This, of course, does not mean that the search for embryo markers, denoting superior embryo quality and pregnancy chances, should be abandoned. Being able to identify embryos with maximal implantation and pregnancy chances, still, represents a "holy grail" of IVF, especially if such markers can be clinically utilized in cost effective ways. The concepts of ES so far pursued in IVF practice, however, do not appear to work efficiently.

In practical terms this means that the clinical utilization of current ES methods should be restricted to investigational use until target patient populations are identified in which ES really does offer clinical outcome benefits. Moreover, new ideas about how ES could be achieved cost-effectively appear urgently needed.
